# Alterations in P-glycoprotein Expression in the Placenta of Obese Rats and Humans

**DOI:** 10.3390/ijms26146976

**Published:** 2025-07-20

**Authors:** Péter Szatmári, Kata Kira Kemény, Andrea Surányi, Yakov Rachamim, Eszter Ducza

**Affiliations:** 1 Department of Pharmacodynamics and Biopharmacy, Faculty of Pharmacy, University of Szeged, Eötvös Street 6, 6720 Szeged, Hungary; szapeti40@gmail.com (P.S.); kemeny.katakira@gmail.com (K.K.K.); 2 Department of Obstetrics and Gynecology, Albert Szent-Györgyi Medical School, University of Szeged, Semmelweis Street 1, 6725 Szeged, Hungary; gaspar-suranyi.andrea@med.u-szeged.hu (A.S.); rachamim.yakov@med.u-szeged.hu (Y.R.)

**Keywords:** P-glycoprotein, ABC transporter, placenta, obesity, rat, human, pregnancy complications, drug exposure

## Abstract

Obesity affects approximately 30% of pregnancies worldwide and is one of the leading metabolic disorders among pregnant women. Maternal obesity is often associated with placental dysfunction and structural alterations, which increase the risk of developing complications. Efflux transporters, including P-glycoprotein (P-gp), may impact placental function and fetal development. Consequently, our research examined the effects of obesity on P-glycoprotein expression in both a rat model and human placental tissue. P-gp expression was measured by RT-PCR and Western blot techniques in human and rat placental tissues. Moreover, we further characterized the high-fat and high-sugar diet (HFHSD)-induced gestational obesity rat model by measuring tissue weights. Significant decreases were observed in fetal, placental, and uterus weights in the obese animals near the end of pregnancy. In obese rats, mRNA and protein expression of placental P-gp showed a reduction on gestation days 15, 20, and 22. A similar P-gp reduction was observed in the term placenta in obese women in mRNA and protein levels. We hypothesize that the reduced expression of P-gp may heighten the susceptibility of both the fetus and placenta to P-gp substrates. This alteration could potentially result in an increased risk of pregnancy complications and obesity-related drug contraindications linked to P-gp transport during pregnancy.

## 1. Introduction

In 2022, approximately 890 million adults (16% of the total adult population) were obese worldwide, increasing year to year [1]. By 2038, an estimated 38% of the population is predicted to be obese globally. Due to the growing prevalence, obesity is one of the leading metabolic disorders among pregnant women and affects ~30% of pregnancies [2,3]. The most common diagnostic parameter of obesity is a body mass index (BMI) of 30 kg/m^2^ or above, according to pre-pregnancy. Significant weight gain occurs during gestation, so the pre-pregnancy BMI is supplemented with optimal weight gain values during pregnancy for a more precise definition of obesity in pregnancy [4].

Obesity is a long-term energy imbalance caused when the calorie consumption exceeds the required utilized energy by the body and results in an excessive body fat accumulation in white adipose tissues through hypertrophy or hyperplasia, both in visceral and subcutaneous regions [5,6,7]. Adipose tissue is not only generated by adipocytes; it is also formed by fibroblasts, endothelial, and immune cells, such as macrophages. Infiltrated macrophages in the adipose tissue produce several pro-inflammatory cytokines [6]. Moreover, adipocytes and other cells of adipose tissue secrete numerous hormonally active substances, namely adipokines like leptin, adiponectin, or visfatin [8]. Most of them are also attributed to pro-inflammatory properties. In parallel, obesity is also associated with triglyceride accumulation in the adipocytes, which increases the β-oxidation of free fatty acids and leads to a high amount of reactive oxygen species (ROS) production in the mitochondria. Moreover, circulating pro-inflammatory cytokines further induce ROS production and vice versa, resulting in oxidative stress and chronic low-grade inflammation [9]. All of these obesity-related mechanisms induce placental dysfunction and structural alterations, which could lead to various pregnancy complications, including gestational diabetes or preeclampsia [2,10]. The placenta is an essential element of in utero development. It produces pregnancy-related hormones and regulates the transport of nutrients, gases, and waste products between the mother and the fetus. Moreover, it protects the fetus from harmful xenobiotics. The main transplacental transport place is the syncytiotrophoblast layer, which separates the maternal blood and fetal circulation and is called the blood–placental barrier. ATP-binding cassette (ABC) transporters are localized in this layer as membrane proteins and mediate the protective mechanisms via the efflux pump function [11]. Among them, ABCB1 (or P-glycoprotein, P-gp) is the best-characterized and studied efflux transporter with a wide range of substrate selectivity, including exogenous compounds like drugs (antidiabetics, antiemetics, antihistamines, etc.), environmental chemicals, dietary constituents or food additives, and endogenous compounds as cytokines, hormones, lipids, or oxidized metabolites [11,12,13]. Beyond the syncytial transporter function, the role of P-gp in the migration, invasion, and differentiation of trophoblast cells for successful placental development is also described [14]. P-gp importance is further emphasized by the International Council for Harmonization M12 guideline, which recommends testing the newly developed products in P-gp substrate and inhibition studies [15]. Drug utilization in pregnancy is common, and in 10% of the cases, drugs with P-gp transport are used. Studies reported that P-gp substrates used during pregnancy in combination with other substrates or inhibitors are associated with an increased risk of congenital anomalies [16], which enlightens us that any change related to P-gp function could result in similar risks. Around the 2000s, studies on placental P-gp expression were the focus when researchers revealed gestational-age-dependent mRNA and protein expression alterations in humans (encoded by the ABCB1 gene) and rodents (encoded by two genes, Abcb1a and Abcb1b) [11]. Emerging data highlight that pathological conditions affect placental P-gp expression; therefore, current researchers focus on the potential influence of diseases during pregnancy [17].

Ethical and technical limitations restrict the investigations on pregnant women. Although human term placental tissues have wide accessibility, placentas from earlier stages of pregnancy are available in a limited number, and a high percentage of them are usually affected by other pathologic states. Hence, the use of rodent models has spread in the field of placental transport research [11].

Recent studies observed a significant correlation between placental P-gp expression alterations and gestational obesity in human and obese mouse models [18,19,20,21]. Wang et al. [18] performed studies similar to ours with C57BL mice. However, the structure and function of the rat and mouse placenta can exhibit similarities; several differences have also been identified between the two species, including the placentation process, protein expression, and the placental transfer of xenobiotics. In our work, we develop a special rat model for the investigation of the effects of obesity on transporter expression. Rats have similar placentation processes as humans, and their gestation period between days 15 and 18 can be considered equivalent to human mid-pregnancy, while gestation day 22 corresponds to human term placenta, which strengthens their human translational potential [22,23]. In our human studies, we plan to confirm the previous results on the Caucasian race [18], which is important for the reproducibility of scientific results and the development of potential new therapeutic targets.

Our main aim was to examine the effect of maternal obesity on placental P-gp expression in a high-fat–high-sugar diet-induced obese rat model in late pregnancy and confirm the animal results with human studies. These studies aimed to create an animal model suitable for the translational investigation of the passage of pharmaceutical compounds through the human placenta in the future.

## 2. Results

### 2.1. Effect of HFHSD on Body Weight and Glucose Tolerance in Rats Before and During Pregnancy

**Figure 1:**
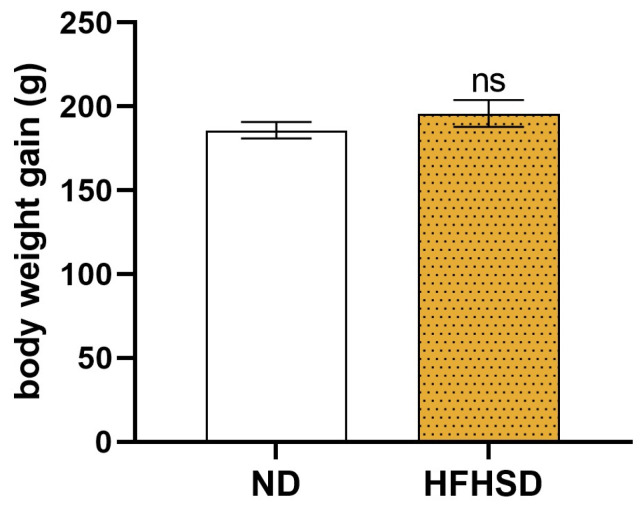
Body weight gain of normal diet (ND) and high-fat–high-sugar diet (HFHSD) rats from the age of four weeks to the beginning of pregnancy. ns: *p* > 0.05, compared to the ND group. *n* = 30 in each diet group.

Before pregnancy, the body weight gain of female rats was higher in the high-fat–high-sugar diet (HFHSD) group. However, it was not statistically significant compared to the normal diet (ND) animals (Figure 1).

**Figure 2:**
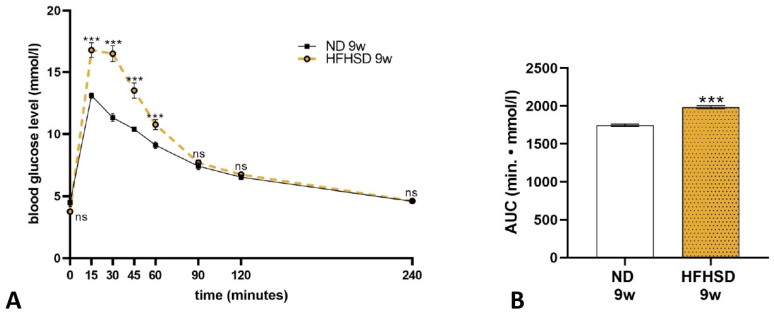
Blood glucose levels (**A**) and area under curve (AUC) analysis (**B**) of normal diet (ND) and high-fat–high-sugar diet (HFHSD) rats at 9 weeks of age. ns, *p* > 0.05; ***, *p* < 0.001, compared to the ND group. *n* = 30 in each diet group.

A glucose tolerance test on 9-week-old female rats revealed that the blood sugar level was significantly increased in the HFHSD rats after 15, 30, 45, and 60 min (Figure 2A). Calculation of the area under the curve (AUC) showed a significant increase in the blood glucose concentration of HFHSD rats compared to the ND group (Figure 2B).

**Figure 3:**
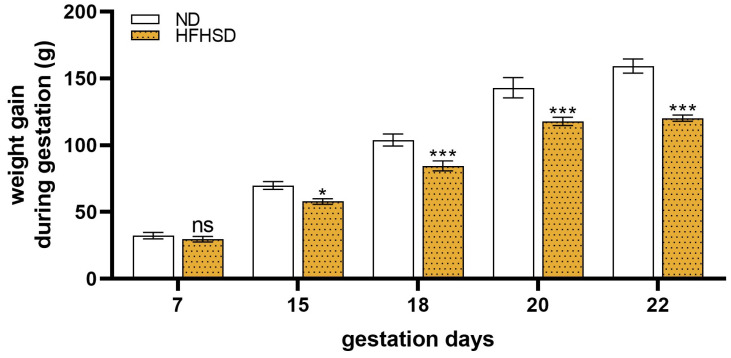
Weight gain of normal diet (ND) and high-fat–high-sugar diet (HFHSD) rats during gestation. ns, *p* > 0.05; *, *p* < 0.05; ***, *p* < 0.001, compared to the ND group. *n* = 6/gestational day in each diet group.

Altered weight gain was observed during pregnancy in the HFHSD-fed rats. Until gestation day 7, no differences were measured between the two diet groups. On gestation days 15, 18, 20, and 22, body weight gain was significantly decreased in the HFHSD dams as compared to the ND group (Figure 3).

**Figure 4:**
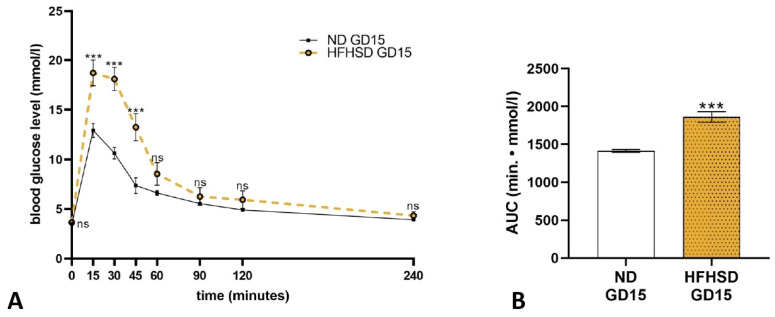
Blood glucose levels (**A**) and area under the curve (AUC) analysis (**B**) of normal diet (ND) and high-fat–high-sugar diet (HFHSD) rats on gestational day 15. ns, *p* > 0.05; ***, *p* < 0.001, compared to the ND group. *n* = 6 in each diet group.

On gestation day 15, the glucose tolerance test indicated that animals subjected to a HFHSD exhibited significantly elevated blood glucose levels at the 15, 30, and 45 min marks in comparison to the ND group (Figure 4A). Furthermore, the AUC value of the glucose plasma curve for the HFHSD dams demonstrated a significant increase relative to that of the ND dams (Figure 4B).

### 2.2. Changes in the Weights of the Uterus, Fetus, and Placenta in HFHSD Rats

**Figure 5:**
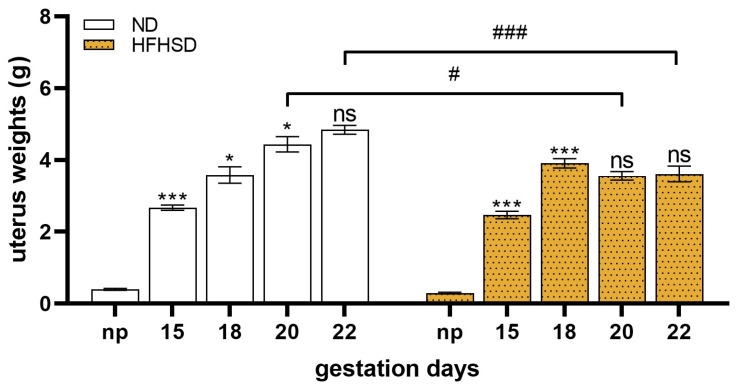
Changes in uterus weights in normal diet (ND) and high-fat–high-sugar diet (HFHSD) in pregnant rats of different gestational ages. np: non-pregnant. ns, *p* > 0.05; *, *p* < 0.05; ***, *p* < 0.001, compared to the previous gestation day. #, *p* < 0.05; ###, *p* < 0.001, compared to the ND group.

In the ND group, the weight of the uterus exhibited a continuous increase throughout the duration of pregnancy. Conversely, in the HFHSD group, the increase was significant until gestation day 18, after which it remained constant until the conclusion of the pregnancy. From gestation day 20, the uterine weights of the HFHSD group were significantly lower compared to ND (Figure 5).

**Figure 6:**
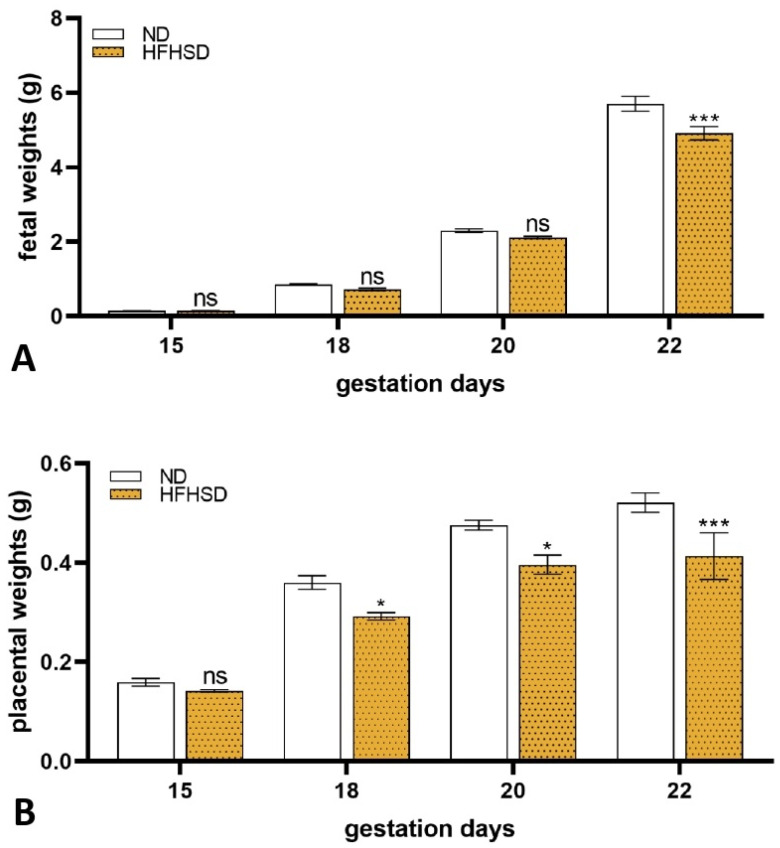
Changes of fetal (**A**) and placental (**B**) weights in normal diet (ND) and high-fat–high-sugar diet (HFHSD) pregnant rats with different gestational ages. ns, *p* > 0.05; *, *p* < 0.05; ***, *p* < 0.001, compared to the ND. Fetal and placental weights are represented by the mean of individual litter values.

Fetal weights were unchanged on gestation days 15, 18, and 20 in both groups, while the 22-day-old pregnant HFHSD-fed rats’ fetuses had significantly lower weights than the ND group (Figure 6A).

Similar alterations were determined in the case of placental weights. No differences between ND and HFHSD animals were determined in placental weight on the 15th day of gestation. Significant decreases were measured on gestation days 18, 20, and 22 in the obese group (Figure 6B).

The average number of implantation sites and the litter size were significantly lower in the HFHSD-fed animals than in the ND rats (Table 1).

### 2.3. Changes in Expression of P-glycoprotein in Rat Placenta

**Figure 7:**
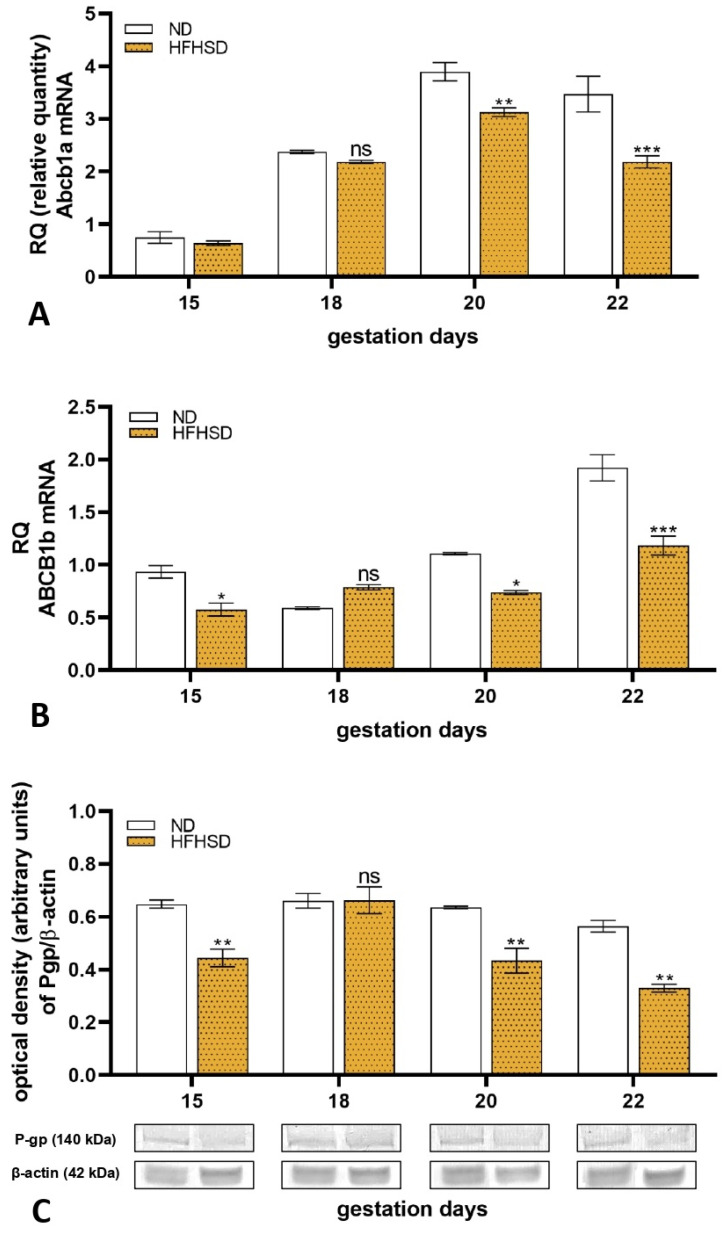
Changes in placental Abcb1a (**A**) and Abcb1b (**B**) mRNA and ABCB1 (**C**) protein expressions in normal diet (ND) and high-fat–high-sugar diet (HFHSD) pregnant rats with different gestational ages. ns, *p* > 0.05; *, *p* < 0.05; **, *p* < 0.01; ***, *p* < 0.001, compared to the ND group. *n* = 6/gestational day in each diet group.

Molecular analysis of the placental tissues showed altered P-gp expression both in mRNA and protein levels between the ND and HFHSD-fed rats during gestation. The mRNA expression of placental Abcb1a was unchanged on the 15th and 18th days of gestation, while it decreased on pregnancy days 20 and 22 in the HFHSD group as compared to the ND group (Figure 7A). The Abcb1b mRNA expression decreased on 15, 20, and 22-day-old HFHSD rat placenta but remained unchanged on day 18 compared to the ND animals (Figure 7B). The protein expression of P-gp was significantly reduced on gestation days 15, 20, and 22 in the obese rats (Figure 7C). The uncropped gel pictures of the Western blot studies are presented in Supplementary Material (Supplementary Figure S1).

### 2.4. Changes in Expression of P-glycoprotein in Human Placenta

**Figure 8:**
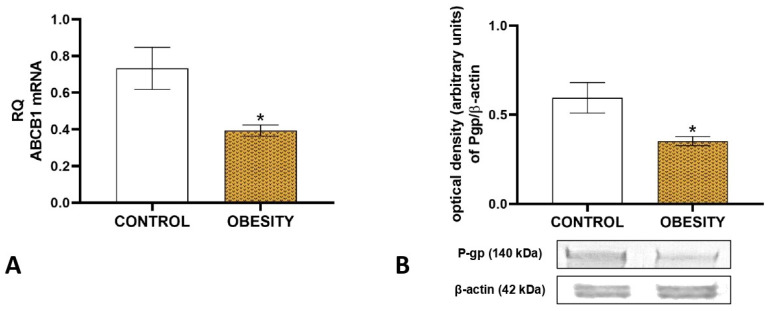
Changes in placental ABCB1 mRNA (**A**) and protein (**B**) expressions in term (pregnancy weeks 40) human obesity-complicated placental tissues. * *p* < 0.05, compared to the control group. *n* = 12 in each group.

Obesity significantly decreased the placental ABCB1 mRNA expression in humans as compared to controls at pregnancy week 40 (Figure 8A). The protein expression of placental P-gp was also reduced significantly in the obese human group compared to the controls (Figure 8B). The uncropped gel pictures of the Western blot studies are presented in Supplementary Material (Supplementary Figure S2).

## 3. Discussion

The medical complications of pregnancies are often associated with impaired placental ABC transporter expressions, placental dysfunctions, or structural alterations [2,17]. However, research on pregnant women is limited by ethical, technical, and regulatory perspectives; alternative approaches are needed [11]. Rodent models are frequently used in the research of reproduction because they show several similarities with human pregnancy and could be manipulated in different ways with nutritional or pharmacological interventions. In addition, rodents have short gestation and lifespans, and tissue collection is accessible during any gestation period, unlike humans [24]. In this study, we examined the effect of gestational obesity induced by HFHSD on the expression of placental P-gp in rat models during late pregnancy and confirmed our animal results with human experiments.

### 3.1. Animal Model

The combination of a high-fat and high-sugar diet results in a slow increase in body weight gain, which takes several weeks, with the early development of glucose intolerance. The induction of obesity with a high-fat and high-sugar combination diet is more similar to human obesity development [25,26]. In our experiment, HFHSD nutrient composition was formed by 28% fat, 16% protein, and 56% carbohydrates. The HFHSD did not lead to a significant increase in weight gain before pregnancy, while their blood glucose levels significantly increased. We suppose that the higher sugar content in our diet restricts the intensive body fat gain during the experiment, which explains the mild elevation in body weight gains and the significantly higher blood glucose levels in our HFHSD animals. The impact of HFHSDs on the weights of maternal, placental, fetal, and uterine tissues on gestation day 22 has been previously documented, along with the observed alterations in glucose tolerance during pregnancies characterized by HFHSD [27]. In current research, we found similar weight reductions of these tissues on gestation day 22. We identified worse glucose tolerance on gestation day 15, the same as previously examined on gestation day 20 [27], which confirms the reproducibility of the model and the presence of a prediabetic state during pregnancy. Similar reductions in weight gains and placental or uterine tissues were observed in the HFHSD animals on other gestation days, which presumably can be explained by the altered metabolic state and the development process of gestation. In the early stages of a healthy human pregnancy, insulin sensitivity is higher. With the progression of pregnancy, a relative insulin resistance state begins to appear with a moderate rise in maternal glucose level and insulin secretion to support the extensive placental and fetal growth in the third trimester [2,28]. In our pregnant animals, we observed a steeper decline in the plasma sugar levels during the glucose tolerance test from 15 min to 120 min than in non-pregnant, which assumes elevated insulin levels with the development of pregnancy. Since the decreased glucose tolerance has already existed in the obese group before pregnancy and it is further worsened during pregnancy, it may be assumed that this alteration interferes with the physiologically increased insulin sensitivity at the early stages of pregnancy and obstructs the higher glucose uptake in the adipose tissue, which may contribute to lower weight gain until pregnancy day 15. After gestation day 15, the major determinants of maternal weight gain are the rates of placental, fetal, and uterine growth. In the case of rodents, the three tissue weights increase with pregnancy progression until term. During the first (before gestation day 7) and second trimester (between gestation days 8–14), tissue weights and maternal body weight gain are slowly growing while placental and fetal weights reach approximately equal values on gestation day 15, then both of them start to significantly grow during the last trimester (after gestation day 15) [29,30,31]. The uterus grows with gestational age until it reaches its plateau weight near the end of the term [32]. All of these physiological weight changes are also observed in our control dams. We suppose that due to the worsened glucose tolerance of obese pregnant animals, placental tissues probably cannot keep in step with the intensive predestined growth after gestation day 15, resulting in placental weight loss. It can be assumed that in obese rats, during the first stages of pregnancy, the placenta grows normally and does not show significant changes near gestation day 15 because it can utilize the glucose by the maternal insulin even in prediabetic conditions. After gestation day 15, a prediabetic condition with elevated glucose levels and glucose intolerance is still present; nevertheless, the growth of the placenta at that time mostly depends on fetal insulin production because of the insulin receptor switch from maternal to the fetal side of the placenta with the progression of pregnancy [28]. For the normal development of the placenta, more insulin should be needed to compensate for obesity-induced insulin resistance. However, fetal insulin secretion response to the higher glucose level is poor in rats during gestation [33]; therefore, it could not provide the appropriate glucose utilization for the placenta, which results in a decrease in placental weight. While fetal growth also depends on its own insulin production, similar weight reduction happens due to immature insulin production response and resistance, in contrast to humans [34,35,36,37]. Next to the placental and fetal weight reductions, the worse glucose tolerance probably also restricts the implantation process, which contributes to the lower litter size of HFHSD dams. Furthermore, the smaller litter size and decreased placental and fetal weights from gestation day 18 presumably contribute to less expansion of the uterus tissue, which also contributes to the lower uterus weights after gestation day 18. It is worth mentioning that diabetes with altered glucose tolerance in rats results in decreased uterus, placental, and fetal weights or numbers [38,39]; therefore, our results further established the presence of a prediabetic condition in our gestational obesity rat model and the relevance of impaired glucose tolerance in obesity. Both the tissue weight reductions and the decreased fetal number probably further limit the excessive weight gain, resulting in a significantly lower body weight of HFHSD rats after gestation day 15. Our HFHSD dams’ weight gain results are correlated with the human recommendation of gestational obesity, while pre-pregnancy obese women are expected to have lower weight gain during pregnancy than normal-weight mothers [4].

### 3.2. Expression of Placental P-glycoprotein in Obesity

Numerous ABC transporter subtypes are identified in the human and rodent placenta [11]; therefore, according to the ICH M12 guideline, we focused on the expression determination of the P-glycoprotein (ABCB1) transporter. Several studies have examined the effect of obesity on the expression of placental P-gp [18,19,20,21]. However, our research was the first to discover the P-gp expression reductions in obese rats during late pregnancy and compare them with human data. Novotna et al. have already revealed that the level of both mRNA isoforms of Abcb1 and protein in rats fed a standard diet increases with the progression of gestation [40]. In our recent study, we found that HFHSD-induced obese rats have lower placental P-gp expression near the end of pregnancy than controls. Both Abcb1a and Abcb1b mRNA expressions showed the same alterations on gestation days 18, 20, and 22 in the HFHSD group. Interestingly, on gestation day 15, expression of Abcb1a remained unchanged, while Abcb1b decreased in the obese animals. P-gp protein expression signifies that both mRNA expression changes contribute to the final protein expression; however, an Abcb1b dominance can also be observed in the obesity-affected rat placenta. These isoform expression discrepancies further established the similar but partly different regulatory pathways and tissue specificity between the two transcripts [19,40,41,42]. It is notable to say that previous studies on diet-induced obese mice in late pregnancy revealed similar placental P-gp expression reductions as in our rats [18,19]. As mentioned above, pregnancy day 22 in rats is correlated with the stage of human term. In our human experiments, we found P-gp reduction both in mRNA and protein levels in the placental tissues, which is consistent with our HFHSD-induced obese rat study related to the P-gp expressions. The effect of obesity on placental P-gp expression was previously described by Wang et al., who determined a similar P-gp decrease in obese term human placenta. However, they performed their experiments on placental tissues by vaginal delivery from gestation age 38–41 weeks and classified obesity at BMI 28 or above in their Chinese individuals [18]. We obtained the same expression patterns with our Caucasian individuals, who were classified as obese at a BMI of 30 or above, with cesarean section at gestation week 40. These results suggest that changes in obesity-induced placental P-gp expression are probably not affected by ethnicity and the mode of delivery. In contrast to this, Scott et al. did not find significant changes in placental ABCB1 expression in overweight or obesity in term human placenta, which may be caused by the antibiotic or glucocorticoid treatment exposure [20]. A recent study explored in humans that obesity increases the mRNA expression of placental ABCB1 before 12 weeks of pregnancy [21]. This lets us hypothesize that obesity-induced expression changes also follow a gestational age-dependent manner. Our conception is further proved by the different expression alterations on different gestation days in our HFHSD-induced obese rats. Considerable evidence is provided that in the obese mouse placenta, digoxin concentration elevation in the fetus is strongly correlated with the obesity-induced P-gp expression reductions during pregnancy [18], which shows that the potential protective function of P-gp is weakened in obesity. Based on this, we suppose that obesity could increase fetal vulnerability against P-gp substrates during pregnancy in both rats and humans. This suggestion is supported by the proof that the inhibition of P-gp by using multiple substrates or inhibitors of this transporter is associated with congenital anomalies compared to their use in monotherapy [16,43]. Moreover, a recent study confirmed that fetal death is associated with decreased placental P-gp expression via local inflammation of the placenta [44]. Obesity is associated with several health problems; therefore, drug utilization is higher than among non-obese patients [45]. Among pregnant women who use some medications, approximately 10% of drugs are associated with P-gp transport [16]. Herbal extracts are commonly used in dietary supplements, and several of them have P-gp inhibitory effects [46]. In addition, among dietary constituents, such as non-nutritive sweeteners or food additives, and environmental contaminants, like microplastics, P-gp inhibitors are also found [47,48,49]. These xenobiotics are detailed in Table 2. Based on our results, we assume that in obesity, a higher substrate concentration could reach the fetal compartment, which may increase fetal and placental complications during pharmacotherapy. Moreover, the inhibitory effects of the various exogenous compounds may further increase the fetal and placental vulnerability in obesity.

It is important to mention that P-gp also transports endogenous compounds like cytokines (IL-1β, IL-6, TNFα) or lipids [11,44]. As described earlier, obesity is associated with low-grade inflammation via increased levels of cytokines. However, oxidative reactions are more intensive due to obesity-related oxidative stress; toxic oxidation products are also produced, and among the oxidized metabolites of lipids, P-gp substrates are present [12,13]. We suppose that circulating cytokines and toxic oxidized substrates could accumulate in the placenta, damage its structure and function, or induce local placental inflammation, leading to fetal and maternal complications. Beyond the substrate transport, P-gp is also involved in the regulation of trophoblast migration, invasion, and differentiation in placental development. Dunk et al. revealed that decreased placental P-gp expression presumably contributes to the development of preeclampsia [14]. Preeclampsia causes are unknown; however, inflammation, oxidative stress, and placental abnormalities are identified as trigger factors and are proven to occur in greater numbers in the case of obesity-complicated pregnancies [50,51]. Since near pregnancy day 15 corresponds with human gestation week 20, and preeclampsia typically occurs after 20 weeks of pregnancy, our hypothesis is that the obesity-induced reduction of placental P-gp expression on gestation day 15 in rats is probably similar in humans near pregnancy week 20, which presumably contributes to the development of preeclampsia in obese mothers via the previously discussed processes.

## 4. Materials and Methods

### 4.1. Housing and Handling of the Animals

All animal experiments were complied with the ARRIVE guidelines and carried out with the approval of the National Scientific Ethical Committee on Animal Experimentation (registration number: IV/911/2025) and following the European Communities Council Directive (2010/63/EU) and the Hungarian Act for the Protection of Animals in Research (Article 32 of Act XXVIII). All experiments were performed in accordance with relevant regulations and guidelines. Male and female Sprague–Dawley rats were procured from INNOVO Ltd. (Gödöllő, Hungary) and were kept in controlled rooms with 22 ± 3 °C temperature, 30–70% relative humidity, and a 12/12 h light/dark period. Rats were mated, and the newborn animals were used in our investigations. At the age of 4 weeks, male and female offspring were randomly classified equally into two groups and maintained on tap water and different diets ad libitum: normal diet (ND) and high-fat, high-sugar diet (HFHSD). The ND group continued to receive a standard diet (Altromin Spezialfutter GmbH & Co. KG, Lage, Germany), while the HFHSD group commenced a high-fat, high-sugar diet (comprising 28% fat, 16% protein, and 56% carbohydrates, C1011, Altromin Spezialfutter GmbH & Co. KG, Lage, Germany) for the duration of the experiment. The female animals were sacrificed under deep isoflurane (AErane liquid for inhalation, Baxter Hungary Ltd., Budapest, Hungary) anesthesia by exsanguination using a portable small animal anesthesia machine (R550, RWD, Shenzhen, China) at the 13th week.

### 4.2. Mating of the Animals

The sexually mature 10-week-old male (240–260 g) and female (200–250 g) rats with the same diet state were mated in a special mating cage in the early morning hours. The day before mating, the estrus cycle was measured in the case of female rats by a Rat Vaginal Impedance Checker (MK-12, Muromachi Kikai Co., Ltd., Tokyo, Japan) between 03:00–04:00 p.m. Rats with vaginal impedance values over 3.0 kΩ are in the proestrus stage and were selected for the mating course for the following morning. Male and female rats were isolated by a movable metal door, which was opened automatically between 03:00–05:00 a.m. and provided the appropriate intercourse until 08:00–09:00 a.m. The existence of pregnancy was determined by vaginal smear examination with a microscope at 20x magnification. Pregnancy was declared by the presence of sperm in the sample or a visible copulation plug in the vagina and considered as the first day of pregnancy. Pregnant animals were separated equally (*n* = 6/pregnancy state) and continued the diet they received before pregnancy until termination. Male animals were only used for mating.

### 4.3. Experimental Design

**Figure 9:**
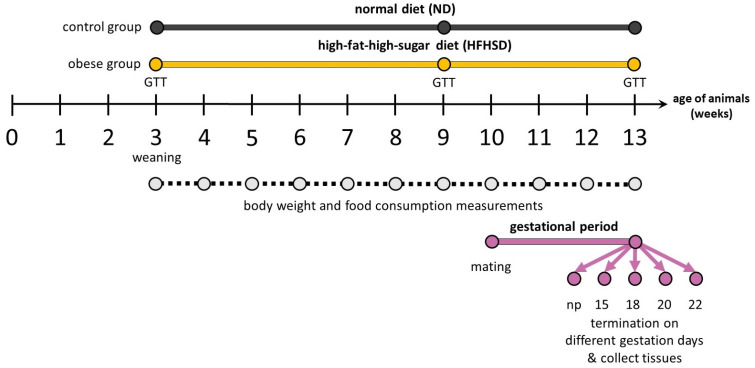
The HFHSD-induced obese animal model experimental design. ○: planned interventions, GTT: glucose tolerance test, np: non-pregnant. Animals were weaned at the age of 21 days (end of 3 weeks of age) and started to be maintained on different diets from the age of 22 days (the beginning of 4 weeks of age).

A total of 60 female rats underwent a ten-week nutritional intervention from 4 weeks of age until 13 weeks (Figure 9). Animals were divided equally into two diet groups. The HFHSD group received a high-fat, high-sugar diet to induce obesity, while the ND group was maintained on standard rodent pellets to serve as a control group. Body mass of the animals and chow consumption were measured every week, including the gestation period, until termination. At 4 and 9 weeks of age, glucose tolerance tests (GTTs) were carried out on all animals. The following week, mature animals were mated at 10 weeks of age, and GTT was measured again in ND and HFHSD groups on 6–6 dams at gestation day 15. At 13 weeks of age, female rats were sacrificed from both diet groups of 6 animals each on gestation days 15, 18, 20, and 22 and in the non-pregnant state. Following termination, the whole uterus and all fetuses and placental tissues were excised, and wet weights were measured. The number of implantation sites of the uterus, resorbed fetuses, and the litter size were also recorded on each gestation day, as previously described [52]. As implantation sites and fetal numbers remain constant after gestation day 15 until delivery [29,30,53], a total mean of each parameter was calculated from all gestation days (*n* = 24 dam/diet group). Due to technical limitations, fetal sex was not determined, and sex-specific alterations were not evaluated. After weight measurement, placental samples were immediately put into RNAlater Solution (Sigma-Aldrich, Budapest, Hungary), frozen in liquid nitrogen, and stored at −80 °C until the molecular studies.

### 4.4. Glucose Tolerance Test

The glucose tolerance test (GTT) was carried out both in ND and HFHSD at week 4, 9, and gestation day 15 (Figure 1). Blood glucose levels were determined with a Dcont^®^ ETALON^®^ Glucose Meter (77 Elektronika Ltd., HU, Budapest, Hungary). The first glucose levels were measured at 08:00 after a 16 h fasting period from one drop of tail vein blood. Following that, rats were injected intraperitoneally with 2 g/kg glucose in the form of a 25% solution. Blood sugar levels were measured at 15, 30, 45, 60, 90, 120, and 240 min after administration. Glucose concentration–time curves were constructed from the measured glucose level at each sampling time, and the area under the curve (AUC) calculation was performed.

### 4.5. Human Samples Collection

A prospective, cross-sectional cohort study was conducted in pregnant women undergoing elective caesarean section at the Department of Obstetrics and Gynecology, University of Szeged, Hungary, between 1 June 2023 and 31 March 2024. During the study period, all singleton pregnancies had an increased risk of fetal death, where a caesarean section was performed at 40 weeks of gestation. Exclusion criteria of the study were identified as follows: multiple pregnancies; fetal or neonatal structural or genetic anomaly; improper localization of the placenta (e.g., placenta praevia); pathological placentation (placenta accreta spectrum); self-reported drug, alcohol, or nicotine abuse.

The determination of gestational age was based on the first day of the last menstrual period and/or on ultrasound biometry (crown–rump length and biparietal diameter) at the 10th week of pregnancy. The characteristics of normal and obese pregnant women were published in our previous study [37].

The placental samples (cc. 1 cm^3^) collection from normal and obese women (BMI  >  30 kg/m^2^) was carried out at the Obstetrics and Gynecology Clinic, University of Szeged. A total of 24 placental samples were collected and investigated from normal (*n* = 12) and obese (*n* = 12) mothers. The human study protocol was approved by the Clinical Research Ethics Committee of the University of Szeged (Ref. no.: 57/2020-SZTE). The study was carried out according to the Principles of the Declaration of Helsinki of 1975. We obtained written informed consent from all participants. The human placental tissues were frozen immediately after collection and stored at −80 °C until assay. All experiments were performed in accordance with relevant regulations and guidelines.

### 4.6. RT-PCR Study

The placental tissues (2–3/dam) were mechanically powdered with a Sartorius MikroDismembrator U (Sartorius, Göttingen, Germany), and the total cellular RNA was extracted by guanidinium thiocyanate acid–phenol–chloroform according to the procedure of Chomczynski and Sacchi [54]. After precipitation with isopropanol, the RNA was washed with 75% ethanol and resuspended in diethyl pyrocarbonate-treated water. RNA purity was controlled at an optical density of 260/280 nm with BioSpec Nano (Shimadzu, Kyoto, Japan); all samples exhibited an absorbance ratio in the range of 1.6–2.0. Reverse transcription and amplification of the PCR products were performed by using the TaqMan RNA-to-CT-Step One Kit (Thermo Fisher Scientific, Budapest, Hungary) and an ABI StepOne Real-Time cycler. Reverse-transcriptase PCR amplifications were performed as follows: at 48 °C for 15 min and at 95 °C for 10 min, followed by 40 cycles at 95 °C for 15 s and at 60 °C for 1 min. The generation of specific PCR products was confirmed by melting curve analysis. The following primers were used: assay ID Rn01639253_m1 for the Abcb1a (rat), Rn01529252_g1 for the Abcb1b (rat), Hs00184500_m1 for the ABCB1 (human), and Rn00667869_m1 for β-actin (rat) or Hs01060665_m1 for β-actin (human) as endogenous control (Thermo Fisher Scientific, Budapest, Hungary). All samples were run in triplicate. The fluorescence intensities of the probes were plotted against PCR cycle number. The amplification cycle displaying the first significant increase in the fluorescence signal was defined as the threshold cycle (CT).

### 4.7. Western Blot Analysis

The placental tissues (2–3/dam) were powdered and homogenised with a solution of RIPA lysis, extraction buffer, and protease inhibitors cocktail. Following protein extraction, the protein concentrations in the supernatant layer were measured with a spectrophotometer, then stored at −80 °C until further use. Then, 50 μg of protein per well was subjected to electrophoresis on 4–12% NuPAGE Bis-Tris Gel in XCell SureLock Mini-Cell Units (Thermo Fisher Scientific, Budapest, Hungary). Proteins were transferred from gels to nitrocellulose membranes, using the iBlot Gel Transfer System (Thermo Fisher Scientific, Budapest, Hungary). Antibody binding was detected with the WesternBreeze Chromogenic Western blot immunodetection kit (Thermo Fisher Scientific, Budapest, Hungary). The blots were incubated on a shaker with MDR1 (141 kDa, 1:300, bs-0563R, Bioss Antibody, Woburn, MA, USA) and β-actin (42 kDa, 1:1000, bs-0061R, Bioss Antibody, Woburn, MA, USA) polyclonal antibody in the blocking buffer. Images were captured with the EDAS290 imaging system (Kodak Ltd., Budapest, Hungary), and the optical density of each immunoreactive band was determined with Kodak 1D Image analysis software(version 4.0). Optical densities were calculated as arbitrary units after local area background subtraction.

### 4.8. Statistical Analysis

Statistical analysis was performed using Prism 9.0 software (Graphpad Software Inc., San Diego, CA, USA). Differences between the two dietary groups, or obese and normal women, were calculated by unpaired *t*-tests, while the effect of nutritional and gestational states on the investigated parameters was assessed using two-way ANOVA, which was followed by Bonferroni correction. All of the results are presented as the mean ± standard error of the mean. Significance was accepted at *p* < 0.05.

## 5. Conclusions

In our study, we observed a correlation between gestational obesity-induced P-gp expression reduction between rat and human placental tissues. We further characterized the gestational obese rat model by extending the tissue weighing on different gestation days to measure gestational weight gain and glucose tolerance. Based on these findings, the rat model could have new potential in the research of placental-related molecular protective mechanism alterations in obesity. Moreover, with the obtained results, we can provide a new approach to the development of obesity-related pregnancy complications, as well as identify new contraindications caused by obesity-associated transporter expression changes, which can contribute to a safer, more effective pharmacotherapy in the presence of obesity during pregnancy.

## Figures and Tables

**Table 1:** Pregnancy outcome parameters of normal diet (ND) and high-fat–high-sugar diet (HFHSD) pregnant rats. Values are the mean ± SD. ns, *p* > 0.05; **, *p* < 0.01, compared to the ND group. *n* = 24 dams in each diet group. Detailed pregnancy outcome parameters on each gestation day are presented in the Supplementary Material (Supplementary Table S1).

Pregnancy Outcome Parameters	ND	HFHSD
average number of implantation sites	14.80 ± 1.25	13.29 ± 1.88 **
average implantation success (%)	97.01 ± 3.82	95.13 ± 8.6 ^ns^
average litter size	14.42 ± 1.44	12.08 ± 2.03 **

**Table 2:** P-glycoprotein substrates and inhibitors.

Category		Xenobiotics	P-gp Modulation	Reference
medicinal drugs	analgesic	paracetamol	substrate	[11,16,43]
	antibiotics	azithromycin	inhibitor	
		clarithromycin	inhibitor	
	antidiabetics	metformin	substrate	
		insulin and analogs	inducer	
	antiemetics	metoclopramide	substrate	
	antihistamines	(levo)cetirizine	substrate	
		(des)loratadine	substrate	
		fexofenadine	substrate	
	antivirals	tenofovir	substrate	
		dolutegravir	substrate	
	cardiac glycosides	digoxin	substrate	
	glucocorticoids	prednisolone	substrate	
		budesonide	substrate	
	H_2_ antagonists	cimetidine	substrate	
		ranitidine	substrate	
herbal extracts	alkaloids	capsaicin (*Capsicum frutescens*)	inhibitor	[46]
	furanocoumarins	bergamottin (*Ginkgo biloba*)	inhibitor	
	flavonoids	catechins, quercetin (*Camellia sinensis*)	inhibitor	
dietary constituents	non-nutritive sweeteners	acesulfame potassium	inhibitor	[47,48]
		sucralose	inhibitor	
	food additives	curcumin piperine	inhibitor inhibitor	
environmental contaminant	microplastic-related chemicals	di-isobutyl phthalate	inhibitor	[49]
		1,1-bis(3,5-di-tert-butyl-2-hydroxyphenyl)ethane	substrate	
		2,2’-methylenebis (4-methyl-6-tert-butylphenol)	inhibitor	

## Data Availability

All data accessed and analyzed in this study are available on request from the corresponding author, Eszter Ducza (ducza.eszter@szte.hu).

## Supplementary Materials

The following supporting information can be downloaded at: https://www.mdpi.com/article/10.3390/ijms26146976/s1.
