# Supplementary material for: Alterations in P-glycoprotein Expression in the Placenta of Obese Rats and Humans
Source: Int J Mol Sci. 2025 Jul 20;26(14):6976. doi: 10.3390/ijms26146976 (PMC12295135; doi:10.3390/ijms26146976)

Alterations in P-glycoprotein Expression in the Placenta of Obese Rats and Humans

Péter Szatmári [1], Kata Kira Kemény [1], Andrea Surányi [2], Yakov Rachamim [2] and Eszter Ducza [1,*]

[1] Department of Pharmacodynamics and Biopharmacy, Faculty of Pharmacy, University of Szeged, Eötvös Street 6, 6720 Szeged, Hungary; szapeti40@gmail.com (P.S.); kemeny.katakira@gmail.com (K.K.K.)

[2] Department of Obstetrics and Gynecology, Albert Szent-Györgyi Medical School, University of Szeged, Semmelweis Street 1, 6725 Szeged, Hungary; gaspar-suranyi.andrea@med.u-szeged.hu (A.S.); rachamim.yakov@med.u-szeged.hu (Y.R.)

* Correspondence: ducza.eszter@szte.hu

Supplementary Table S1. Pregnancy outcome parameters of normal diet (ND) and high-fat-high-sugar diet (HFHSD) pregnant rats on each gestation days (n = 6 dam/gestation day). Values are number of mean ± SD. ns $p>0.05$, * $p<0.05$, ** $p<0.01$, compared to the ND group.

**Pregnancy outcome parameters**	**ND**	**HFHSD**
Gestation day 15		
number of implantation sites	14.833 ± 0.753	13.667 ± 1.033 *
number of resorpted fetuses	0.5 ± 0.548	1.333 ± 1.033 ns
litter size	14.333 ± 1.033	12.333 ± 1.366 *
Gestation day 18		
number of implantation sites	14.833 ± 1.722	15.667 ± 0.516 ns
number of resorpted fetuses	0.5 ± 0.548	0.333 ± 0.516 ns
litter size	14.5 ± 1.378	15.333 ± 0.516 ns
Gestation day 20		
number of implantation sites	14.833 ± 1.835	12.167 ± 0.753 **
number of resorpted fetuses	0.333 ± 0.516	0.333 ± 0.816 ns
litter size	14.5 ± 2.258	11.833 ± 0.753 *
Gestation day 22		
number of implantation sites	14.667 ± 0.516	11.667 ± 1.633 **
number of resorpted fetuses	0.333 ± 0.816	0.333 ± 0.816 ns
litter size	14.333 ± 1.211	11.333 ± 2.160 *
Gestation days 15-22		
average number of implantation sites[a]	14.792 ± 1.250	13.292 ± 1.876 **
average number of resorpted fetuses[a]	0.417 ± 0.584	0.583 ± 0.881 ns
average litter size[a]	14.417 ± 1.442	12.078 ± 2.032 **

[a]: presented in the text as Table 1.

Supplementary Figure S1. Changes of P-glycoprotein expression in normal diet (ND) (**A**) and high-fat-high-sugar diet (HF) (**B**) fed rats on different gestation days. Both gels were run in paralel with the same conditions.

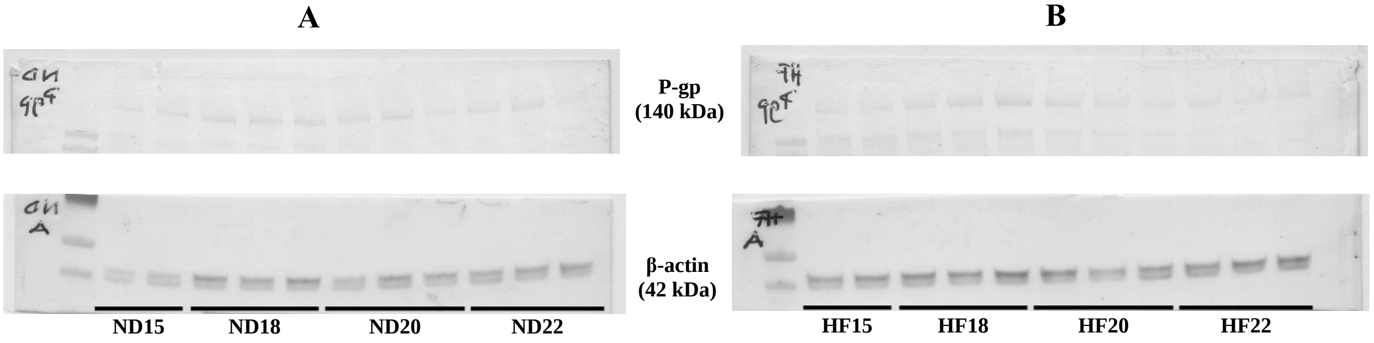

Supplementary Figure S2. Changes of P-glycoprotein expression in normal (Control) and obesity-complicated human term placental tissues. Both gels were run in paralel with the same conditions.

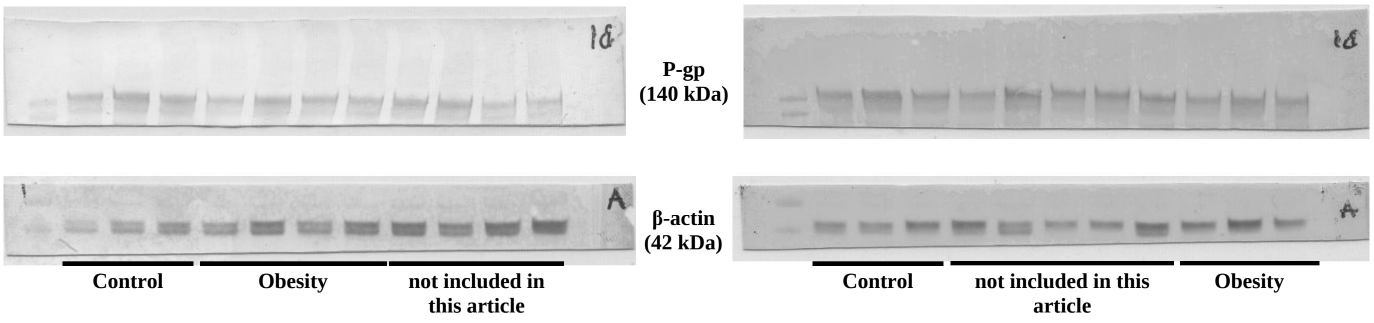

Supplement: Supplementary file 1 [file ijms-26-06976-s001.zip › ijms-3734746-supplementary.pdf]
